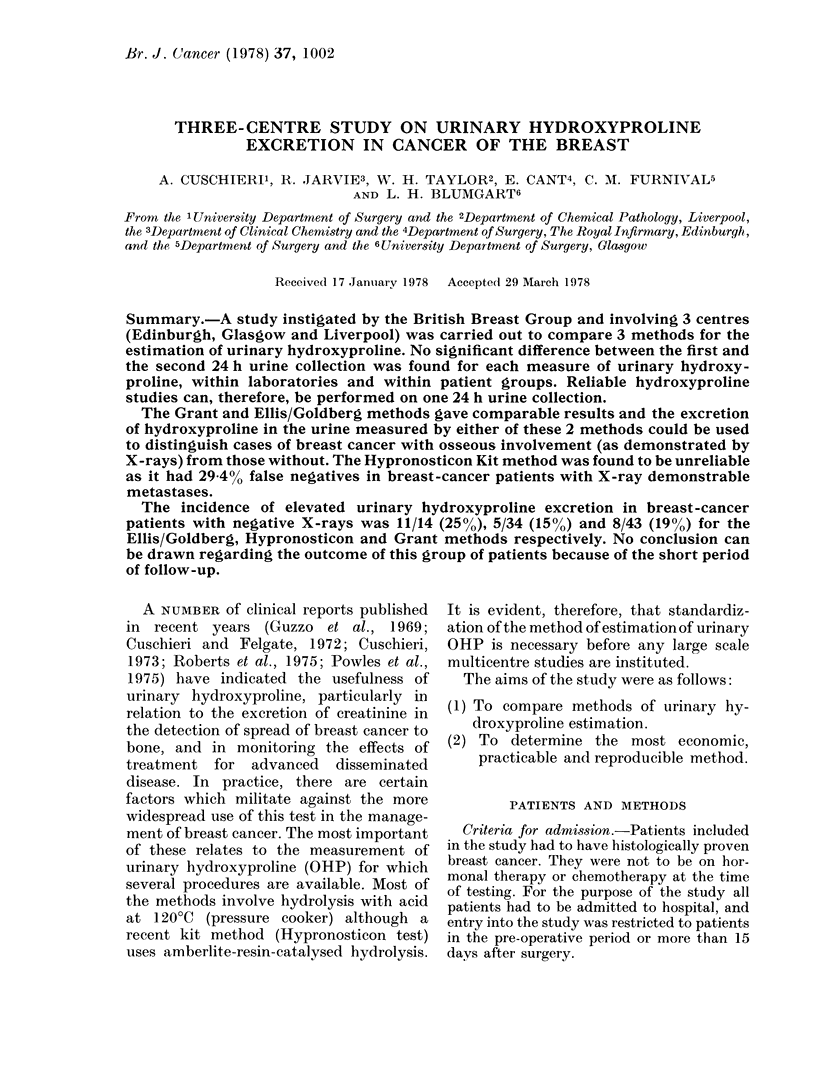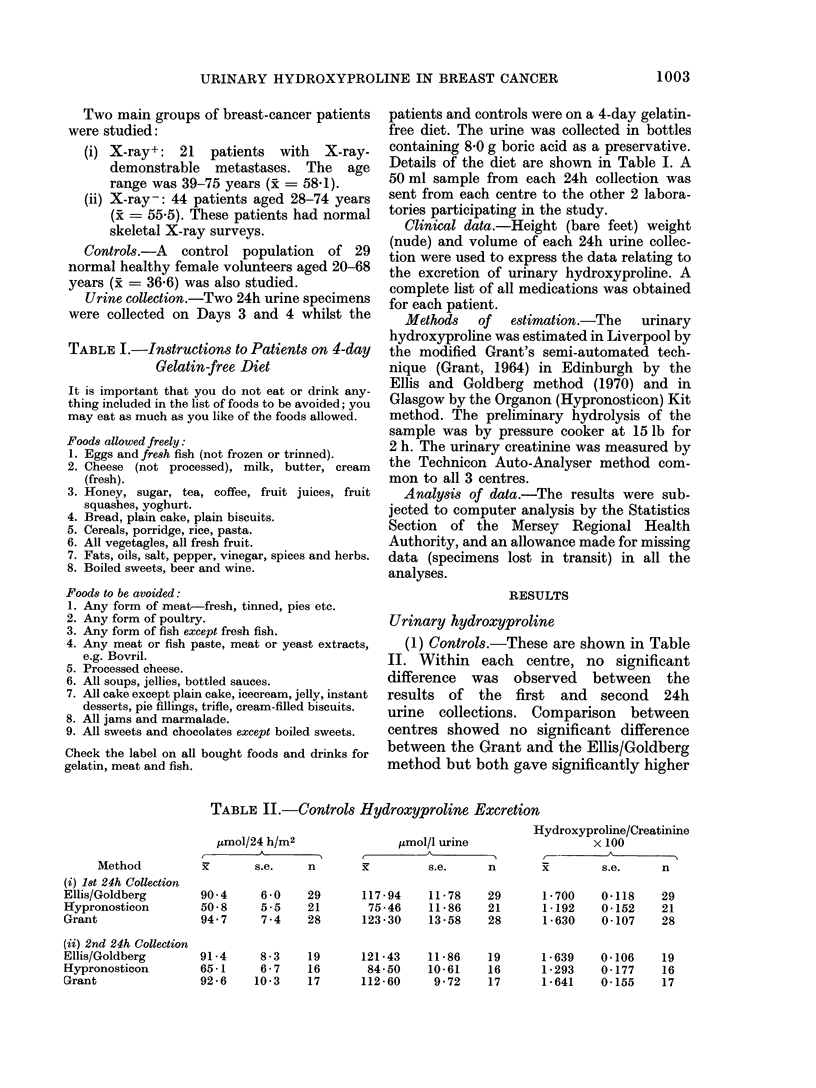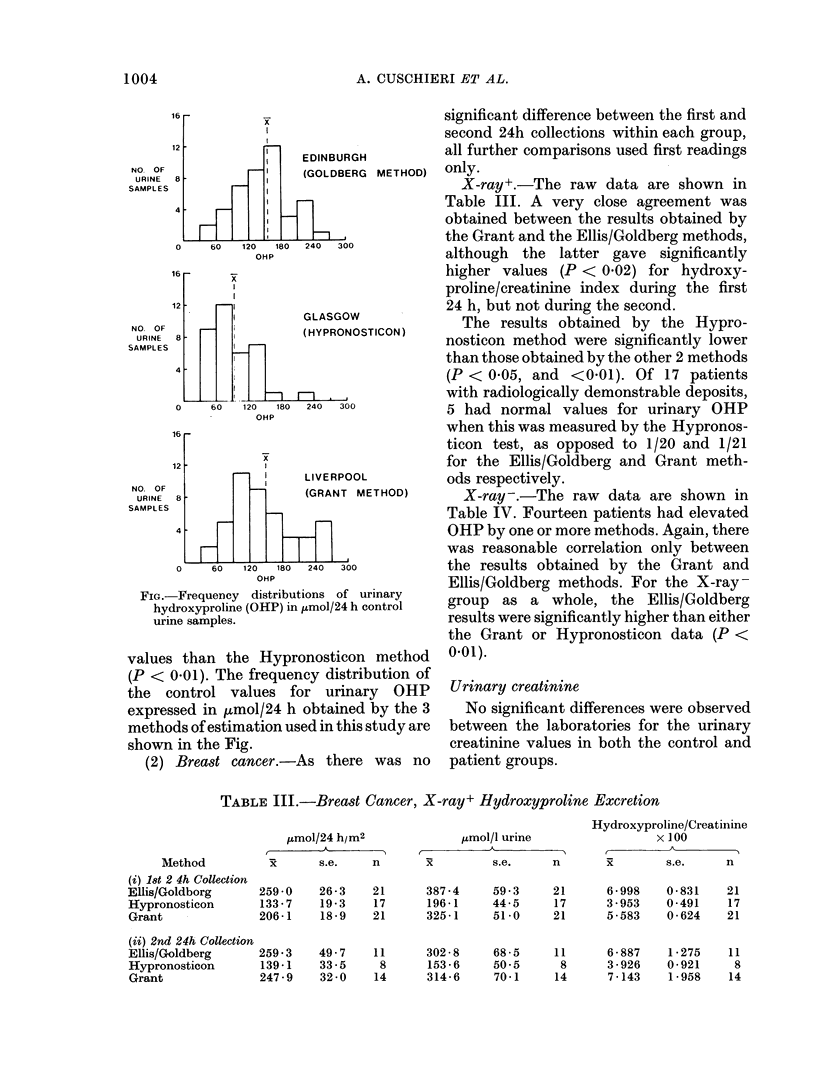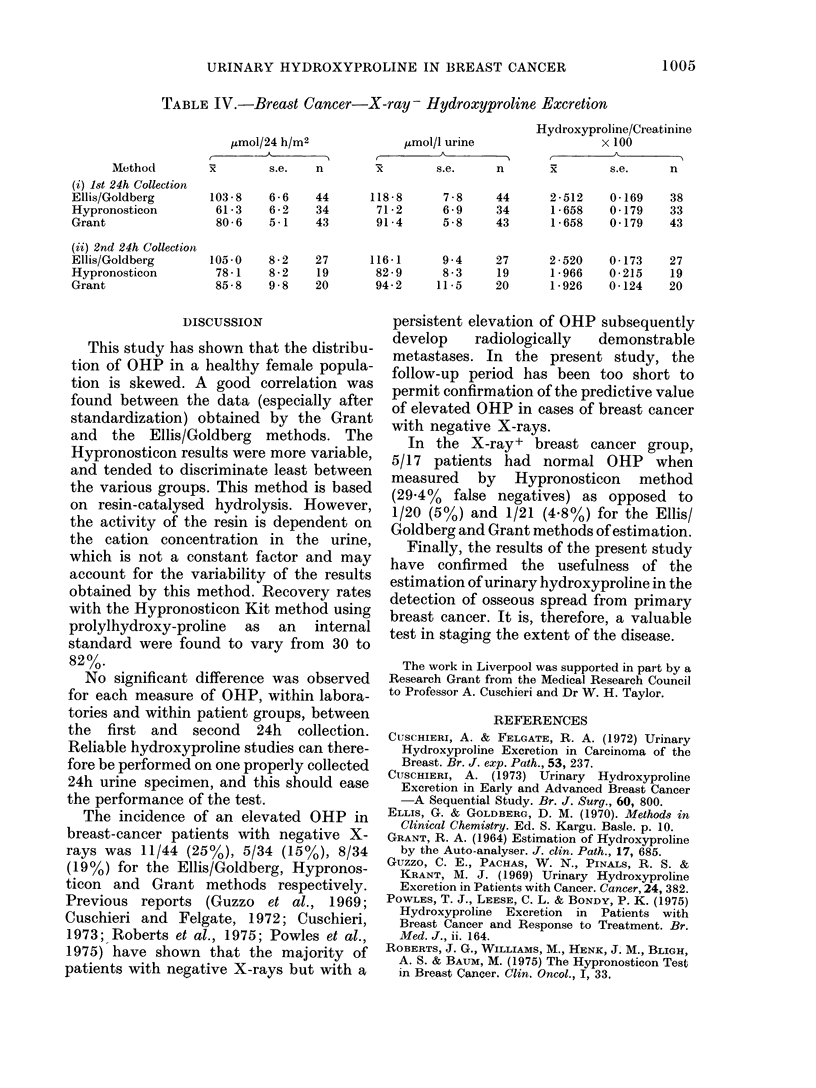# Three-centre study on urinary hydroxyproline excretion in cancer of the breast.

**DOI:** 10.1038/bjc.1978.145

**Published:** 1978-06

**Authors:** A. Cuschieri, R. Jarvie, W. H. Taylor, E. Cant, C. M. Furnival, L. H. Blumgart

## Abstract

A study instigated by the British Breast Group and involving 3 centres (Edinburgh, Glasgow and Liverpool) was carried out to compare 3 methods for the estimation of urinary hydroxyproline. No significant difference between the first and the second 24 h urine collection was found for each measure of urinary hydroxyproline, within laboratories and within patient groups. Reliable hydroxyproline studies can, therefore, be performed on one 24 h urine collection. The Grant and Ellis/Goldberg methods gave comparable results and the excretion of hydroxyproline in the urine measured by either of these 2 methods could be used to distinguish cases of breast cancer with osseous involvement (as demonstrated by X-rays) from those without. The Hypronosticon Kit method was found to be unreliable as it has 29.4% false negatives in breast-cancer patients with X-ray demonstrable metastases. The incidence of elevated urinary hydroxyproline excretion in breast-cancer patients with negative X-rays was 11/14 (25%), 5/34 (15%) and 8/43 (19%) for the Ellis/Goldberg, Hypronosticon and Grant methods respectively. No conclusion can be drawn regarding the outcome of this group of patients because of the short period of follow-up.


*Br. J. Cancer* (1978) **37**, 1002

### THREE-CENTRE STUDY ON URINARY HYDROXYPROLINE EXCRETION IN CANCER OF THE BREAST

A. CUSCHIERI[1], R. JARVIE[3], W. H. TAYLOR[2], E. CANT[4], C. M. FURNIVAL[5] AND L. H. BLUMGART[6]

*From the* [1]*University Department of Surgery and the* [2]*Department of Chemical Pathology, Liverpool, the* [3]*Department of Clinical Chemistry and the* [4]*Department of Surgery, The Royal Infirmary, Edinburgh, and the* [5]*Department of Surgery and the* [6]*University Department of Surgery, Glasgow*

Received 17 January 1978 Accepted 29 March 1978

**Summary.—A study instigated by the British Breast Group and involving 3 centres (Edinburgh, Glasgow and Liverpool) was carried out to compare 3 methods for the estimation of urinary hydroxyproline. No significant difference between the first and the second 24 h urine collection was found for each measure of urinary hydroxyproline, within laboratories and within patient groups. Reliable hydroxyproline studies can, therefore, be performed on one 24 h urine collection.**

**The Grant and Ellis/Goldberg methods gave comparable results and the excretion of hydroxyproline in the urine measured by either of these 2 methods could be used to distinguish cases of breast cancer with osseous involvement (as demonstrated by X-rays) from those without. The Hypronosticon Kit method was found to be unreliable as it had 29·4% false negatives in breast-cancer patients with X-ray demonstrable metastases.**

**The incidence of elevated urinary hydroxyproline excretion in breast-cancer patients with negative X-rays was 11/14 (25%), 5/34 (15%) and 8/43 (19%) for the Ellis/Goldberg, Hypronosticon and Grant methods respectively. No conclusion can be drawn regarding the outcome of this group of patients because of the short period of follow-up.**

A NUMBER of clinical reports published in recent years (Guzzo *et al.*, 1969; Cuschieri and Felgate, 1972; Cuschieri, 1973; Roberts *et al.*, 1975; Powles *et al.*, 1975) have indicated the usefulness of urinary hydroxyproline, particularly in relation to the excretion of creatinine in the detection of spread of breast cancer to bone, and in monitoring the effects of treatment for advanced disseminated disease. In practice, there are certain factors which militate against the more widespread use of this test in the management of breast cancer. The most important of these relates to the measurement of urinary hydroxyproline (OHP) for which several procedures are available. Most of the methods involve hydrolysis with acid at 120°C (pressure cooker) although a recent kit method (Hypronosticon test) uses amberlite-resin-catalysed hydrolysis. It is evident, therefore, that standardization of the method of estimation of urinary OHP is necessary before any large scale multicentre studies are instituted.

The aims of the study were as follows:

(1) To compare methods of urinary hydroxyproline estimation.
(2) To determine the most economic, practicable and reproducible method.

### PATIENTS AND METHODS

*Criteria for admission.*—Patients included in the study had to have histologically proven breast cancer. They were not to be on hormonal therapy or chemotherapy at the time of testing. For the purpose of the study all patients had to be admitted to hospital, and entry into the study was restricted to patients in the pre-operative period or more than 15 days after surgery.

Two main groups of breast-cancer patients were studied:

(i) X-ray+: 21 patients with X-ray-demonstrable metastases. The age range was 39–75 years ($\bar{x}$ = 58·1).

(ii) X-ray−: 44 patients aged 28–74 years ($\bar{x}$ = 55·5). These patients had normal skeletal X-ray surveys.

*Controls.*—A control population of 29 normal healthy female volunteers aged 20–68 years ($\bar{x}$ = 36·6) was also studied.

*Urine collection.*—Two 24h urine specimens were collected on Days 3 and 4 whilst the patients and controls were on a 4-day gelatin-free diet. The urine was collected in bottles containing 8·0 g boric acid as a preservative. Details of the diet are shown in Table I. A 50 ml sample from each 24h collection was sent from each centre to the other 2 laboratories participating in the study.

TABLE I.—*Instructions to Patients on 4-day Gelatin-free Diet*

It is important that you do not eat or drink anything included in the list of foods to be avoided; you may eat as much as you like of the foods allowed.

*Foods allowed freely:*
1. Eggs and *fresh* fish (not frozen or trinned).
2. Cheese (not processed), milk, butter, cream (fresh).
3. Honey, sugar, tea, coffee, fruit juices, fruit squashes, yoghurt.
4. Bread, plain cake, plain biscuits.
5. Cereals, porridge, rice, pasta.
6. All vegetagles, all fresh fruit.
7. Fats, oils, salt, pepper, vinegar, spices and herbs.
8. Boiled sweets, beer and wine.

*Foods to be avoided:*
1. Any form of meat—fresh, tinned, pies etc.
2. Any form of poultry.
3. Any form of fish *except* fresh fish.
4. Any meat or fish paste, meat or yeast extracts, e.g. Bovril.
5. Processed cheese.
6. All soups, jellies, bottled sauces.
7. All cake except plain cake, icecream, jelly, instant desserts, pie fillings, trifle, cream-filled biscuits.
8. All jams and marmalade.
9. All sweets and chocolates *except* boiled sweets.

Check the label on all bought foods and drinks for gelatin, meat and fish.

*Clinical data.*—Height (bare feet) weight (nude) and volume of each 24h urine collection were used to express the data relating to the excretion of urinary hydroxyproline. A complete list of all medications was obtained for each patient.

*Methods of estimation.*—The urinary hydroxyproline was estimated in Liverpool by the modified Grant's semi-automated technique (Grant, 1964) in Edinburgh by the Ellis and Goldberg method (1970) and in Glasgow by the Organon (Hypronosticon) Kit method. The preliminary hydrolysis of the sample was by pressure cooker at 15 lb for 2 h. The urinary creatinine was measured by the Technicon Auto-Analyser method common to all 3 centres.

*Analysis of data.*—The results were subjected to computer analysis by the Statistics Section of the Mersey Regional Health Authority, and an allowance made for missing data (specimens lost in transit) in all the analyses.

### RESULTS

### *Urinary hydroxyproline*

(1) *Controls.*—These are shown in Table II. Within each centre, no significant difference was observed between the results of the first and second 24h urine collections. Comparison between centres showed no significant difference between the Grant and the Ellis/Goldberg method but both gave significantly higher

TABLE II.—*Controls Hydroxyproline Excretion*

| | μmol/24 h/m² | | | μmol/l urine | | | Hydroxyproline/Creatinine × 100 | | |
|---|---|---|---|---|---|---|---|---|---|
| Method | $\bar{x}$ | s.e. | n | $\bar{x}$ | s.e. | n | $\bar{x}$ | s.e. | n |
| (*i*) *1st 24h Collection* | | | | | | | | | |
| Ellis/Goldberg | 90·4 | 6·0 | 29 | 117·94 | 11·78 | 29 | 1·700 | 0·118 | 29 |
| Hypronosticon | 50·8 | 5·5 | 21 | 75·46 | 11·86 | 21 | 1·192 | 0·152 | 21 |
| Grant | 94·7 | 7·4 | 28 | 123·30 | 13·58 | 28 | 1·630 | 0·107 | 28 |
| (*ii*) *2nd 24h Collection* | | | | | | | | | |
| Ellis/Goldberg | 91·4 | 8·3 | 19 | 121·43 | 11·86 | 19 | 1·639 | 0·106 | 19 |
| Hypronosticon | 65·1 | 6·7 | 16 | 84·50 | 10·61 | 16 | 1·293 | 0·177 | 16 |
| Grant | 92·6 | 10·3 | 17 | 112·60 | 9·72 | 17 | 1·641 | 0·155 | 17 |

FIG.—Frequency distributions of urinary hydroxyproline (OHP) in $\mu$mol/24 h control urine samples.

values than the Hypronosticon method ($P < 0{\cdot}01$). The frequency distribution of the control values for urinary OHP expressed in $\mu$mol/24 h obtained by the 3 methods of estimation used in this study are shown in the Fig.

(2) *Breast cancer*.—As there was no significant difference between the first and second 24h collections within each group, all further comparisons used first readings only.

*X-ray*$^+$.—The raw data are shown in Table III. A very close agreement was obtained between the results obtained by the Grant and the Ellis/Goldberg methods, although the latter gave significantly higher values ($P < 0{\cdot}02$) for hydroxyproline/creatinine index during the first 24 h, but not during the second.

The results obtained by the Hypronosticon method were significantly lower than those obtained by the other 2 methods ($P < 0{\cdot}05$, and $< 0{\cdot}01$). Of 17 patients with radiologically demonstrable deposits, 5 had normal values for urinary OHP when this was measured by the Hypronosticon test, as opposed to 1/20 and 1/21 for the Ellis/Goldberg and Grant methods respectively.

*X-ray*$^-$.—The raw data are shown in Table IV. Fourteen patients had elevated OHP by one or more methods. Again, there was reasonable correlation only between the results obtained by the Grant and Ellis/Goldberg methods. For the X-ray$^-$ group as a whole, the Ellis/Goldberg results were significantly higher than either the Grant or Hypronosticon data ($P < 0{\cdot}01$).

### *Urinary creatinine*

No significant differences were observed between the laboratories for the urinary creatinine values in both the control and patient groups.

TABLE III.—*Breast Cancer, X-ray*$^+$ *Hydroxyproline Excretion*

| | $\mu$mol/24 h/m$^2$ | | | $\mu$mol/l urine | | | Hydroxyproline/Creatinine × 100 | | |
|---|---|---|---|---|---|---|---|---|---|
| Method | $\bar{x}$ | s.e. | n | $\bar{x}$ | s.e. | n | $\bar{x}$ | s.e. | n |
| (*i*) *1st 2 4h Collection* | | | | | | | | | |
| Ellis/Goldborg | 259·0 | 26·3 | 21 | 387·4 | 59·3 | 21 | 6·998 | 0·831 | 21 |
| Hypronosticon | 133·7 | 19·3 | 17 | 196·1 | 44·5 | 17 | 3·953 | 0·491 | 17 |
| Grant | 206·1 | 18·9 | 21 | 325·1 | 51·0 | 21 | 5·583 | 0·624 | 21 |
| (*ii*) *2nd 24h Collection* | | | | | | | | | |
| Ellis/Goldberg | 259·3 | 49·7 | 11 | 302·8 | 68·5 | 11 | 6·887 | 1·275 | 11 |
| Hypronosticon | 139·1 | 33·5 | 8 | 153·6 | 50·5 | 8 | 3·926 | 0·921 | 8 |
| Grant | 247·9 | 32·0 | 14 | 314·6 | 70·1 | 14 | 7·143 | 1·958 | 14 |

TABLE IV.—*Breast Cancer—X-ray⁻ Hydroxyproline Excretion*

| Method | μmol/24 h/m² | | | μmol/l urine | | | Hydroxyproline/Creatinine × 100 | | |
|---|---|---|---|---|---|---|---|---|---|
| | x̄ | s.e. | n | x̄ | s.e. | n | x̄ | s.e. | n |
| *(i) 1st 24h Collection* | | | | | | | | | |
| Ellis/Goldberg | 103·8 | 6·6 | 44 | 118·8 | 7·8 | 44 | 2·512 | 0·169 | 38 |
| Hypronosticon | 61·3 | 6·2 | 34 | 71·2 | 6·9 | 34 | 1·658 | 0·179 | 33 |
| Grant | 80·6 | 5·1 | 43 | 91·4 | 5·8 | 43 | 1·658 | 0·179 | 43 |
| *(ii) 2nd 24h Collection* | | | | | | | | | |
| Ellis/Goldberg | 105·0 | 8·2 | 27 | 116·1 | 9·4 | 27 | 2·520 | 0·173 | 27 |
| Hypronosticon | 78·1 | 8·2 | 19 | 82·9 | 8·3 | 19 | 1·966 | 0·215 | 19 |
| Grant | 85·8 | 9·8 | 20 | 94·2 | 11·5 | 20 | 1·926 | 0·124 | 20 |

### DISCUSSION

This study has shown that the distribution of OHP in a healthy female population is skewed. A good correlation was found between the data (especially after standardization) obtained by the Grant and the Ellis/Goldberg methods. The Hypronosticon results were more variable, and tended to discriminate least between the various groups. This method is based on resin-catalysed hydrolysis. However, the activity of the resin is dependent on the cation concentration in the urine, which is not a constant factor and may account for the variability of the results obtained by this method. Recovery rates with the Hypronosticon Kit method using prolylhydroxy-proline as an internal standard were found to vary from 30 to 82%.

No significant difference was observed for each measure of OHP, within laboratories and within patient groups, between the first and second 24h collection. Reliable hydroxyproline studies can therefore be performed on one properly collected 24h urine specimen, and this should ease the performance of the test.

The incidence of an elevated OHP in breast-cancer patients with negative X-rays was 11/44 (25%), 5/34 (15%), 8/34 (19%) for the Ellis/Goldberg, Hypronosticon and Grant methods respectively. Previous reports (Guzzo *et al.*, 1969; Cuschieri and Felgate, 1972; Cuschieri, 1973; Roberts *et al.*, 1975; Powles *et al.*, 1975) have shown that the majority of patients with negative X-rays but with a persistent elevation of OHP subsequently develop radiologically demonstrable metastases. In the present study, the follow-up period has been too short to permit confirmation of the predictive value of elevated OHP in cases of breast cancer with negative X-rays.

In the X-ray⁺ breast cancer group, 5/17 patients had normal OHP when measured by Hypronosticon method (29·4% false negatives) as opposed to 1/20 (5%) and 1/21 (4·8%) for the Ellis/Goldberg and Grant methods of estimation.

Finally, the results of the present study have confirmed the usefulness of the estimation of urinary hydroxyproline in the detection of osseous spread from primary breast cancer. It is, therefore, a valuable test in staging the extent of the disease.

The work in Liverpool was supported in part by a Research Grant from the Medical Research Council to Professor A. Cuschieri and Dr W. H. Taylor.

### REFERENCES

CUSCHIERI, A. & FELGATE, R. A. (1972) Urinary Hydroxyproline Excretion in Carcinoma of the Breast. *Br. J. exp. Path.*, **53**, 237.

CUSCHIERI, A. (1973) Urinary Hydroxyproline Excretion in Early and Advanced Breast Cancer —A Sequential Study. *Br. J. Surg.*, **60**, 800.

ELLIS, G. & GOLDBERG, D. M. (1970). *Methods in Clinical Chemistry*. Ed. S. Kargu. Basle. p. 10.

GRANT, R. A. (1964) Estimation of Hydroxyproline by the Auto-analyser. *J. clin. Path.*, **17**, 685.

GUZZO, C. E., PACHAS, W. N., PINALS, R. S. & KRANT, M. J. (1969) Urinary Hydroxyproline Excretion in Patients with Cancer. *Cancer*, **24**, 382.

POWLES, T. J., LEESE, C. L. & BONDY, P. K. (1975) Hydroxyproline Excretion in Patients with Breast Cancer and Response to Treatment. *Br. Med. J.*, ii. 164.

ROBERTS, J. G., WILLIAMS, M., HENK, J. M., BLIGH, A. S. & BAUM, M. (1975) The Hypronosticon Test in Breast Cancer. *Clin. Oncol.*, I, 33.